# Mitochondrial HMG-CoA Synthase Deficiency in Vietnamese Patients

**DOI:** 10.3390/ijms26041644

**Published:** 2025-02-14

**Authors:** Khanh Ngoc Nguyen, Tran Minh Dien, Thi Bich Ngoc Can, Bui Phuong Thao, Tien Son Do, Thi Kim Giang Dang, Ngoc Lan Nguyen, Van Khanh Tran, Thuy Thu Nguyen, Tran Thi Quynh Trang, Le Thi Phuong, Phan Long Nguyen, Thinh Huy Tran, Nguyen Huu Tu, Chi Dung Vu

**Affiliations:** 1 Center of Endocrinology, Metabolism, Genetic/Genomics and Molecular Therapy, Vietnam National Children’s Hospital, 18/879 La Thanh, Dong Da, Hanoi 11512, Vietnam; khanhnn@nch.gov.vn (K.N.N.); ngocctb@nch.gov.vn (T.B.N.C.); thaobp@nch.gov.vn (B.P.T.); dotienson@nch.gov.vn (T.S.D.); giangdk@nch.gov.vn (T.K.G.D.); 2 Hanoi Medical University, 1st Ton That Tung Street, Hanoi 11521, Vietnam; nguyenhuutu@hmu.edu.vn; 3 Vietnam National Children’s Hospital, 18/879 La Thanh, Dong Da, Hanoi 11512, Vietnam; dientm@nch.gov.vn; 4 Center for Gene and Protein Research, Hanoi Medical University, 1st Ton That Tung Street, Hanoi 11521, Vietnam; nguyenngoclan@hmu.edu.vn (N.L.N.); tranvankhanh@hmu.edu.vn (V.K.T.); nguyenthuthuy@hmu.edu.vn (T.T.N.); tranthiquynhtrang@hmu.edu.vn (T.T.Q.T.); phuongle@hmu.edu.vn (L.T.P.); phanlongnguyen1998@gmail.com (P.L.N.); 5 Biochemistry Department, Hanoi Medical University, 1st Ton That Tung Street, Hanoi 11521, Vietnam; tranhuythinh@hmu.edu.vn

**Keywords:** mitochondrial 3-hydroxy-3-methylglutaryl-CoA synthase deficiency, *HMGCS2* variant, Vietnamese, p.D136V, c.559+1G>A, p.F364I, asymptomatic

## Abstract

Mitochondrial 3-hydroxy-3-methylglutaryl-CoA synthase deficiency (HMGCS2D) is a rare metabolic disorder that impairs the body’s ability to produce ketone bodies and regulate energy metabolism. Diagnosing HMGCS2D is challenging because patients typically remain asymptomatic unless they experience fasting or illness. Due to the absence of reliable biochemical markers, genetic testing has become the definitive method for diagnosing HMGCS2D. This study included 19 patients from 14 unrelated families diagnosed with HMGCS2D in our department between October 2018 and October 2024. The clinical presentations, biochemical findings, molecular characteristics, and management strategies were systematically summarized and analyzed. Of the 19 cases studied, 16 were symptomatic, and 3 were asymptomatic. The onset of the first acute episode occurred between 10 days and 28 months of age. Triggers for the initial crisis in the symptomatic cases included poor feeding (93.8%), vomiting (56.3%), diarrhea (25.0%), and fever (18.8%). Clinical manifestations during the first episode were lethargy/coma (81.3%), rapid breathing (68.8%), hepatomegaly (56.3%), shock (37.5%), and seizures (18.8%). The biochemical abnormalities observed included elevated plasma transaminases (100%), metabolic acidosis (75%), hypoglycemia (56.3%), and elevated plasma ammonia levels (31.3%). Additionally, low free carnitine levels were found in seven cases, elevated C2 levels were found in one case, dicarboxylic aciduria was found in two cases, and ketonuria was found in two cases. Abnormal brain MRI findings were detected in three patients. Genetic analysis revealed seven *HMGCS2* gene variants across the 19 cases. Notably, a novel variant, c.407A>T (p.D136V), was identified and has not been reported in any existing databases. Two common variants, c.559+1G>A and c.1090T>A (p.F364I), were present in 11 out of 19 cases (57.9%) and 10 out of 19 cases (55.5%), respectively. The implementation of a high glucose infusion and proactive management strategies—such as preventing prolonged fasting and providing enteral carbohydrate/glucose infusion during illness—effectively reduced the rate of acute relapses following accurate diagnosis. Currently, all 19 patients are alive, with ages ranging from 5 months to 14 years, and exhibit normal physical development. To the best of our knowledge, this study represents the first reported cases of HMGCS2D in Vietnamese patients. Our findings contribute to a broader understanding of the clinical phenotype and expand the known spectrum of *HMGCS2* gene variants, enhancing current knowledge of this rare metabolic disorder.

## 1. Introduction

Mitochondrial 3-hydroxy-3-methylglutaryl-CoA synthase deficiency (HMGCS2D, OMIM #605911) is a rare metabolic disorder that impairs the body’s ability to produce ketone bodies and regulate energy metabolism, particularly during fasting or illness [1]. This condition is caused by a deficiency of the HMG-CoA synthase enzyme, which plays a key role in the ketogenesis pathway by catalyzing the formation of 3-hydroxy-3-methylglutaryl-CoA (HMG-CoA) from acetyl-CoA and acetoacetyl-CoA [2]. This step is essential for the synthesis of acetoacetate and its derivatives, 3-hydroxybutyric acid and acetone, in the liver. Ketone bodies act as an alternative energy source for the brain and muscles when glucose is scarce, such as during fasting or prolonged physical activity [3]. In the absence of the HMG-CoA enzyme, the body cannot effectively produce ketone bodies and becomes entirely dependent on glucose for energy [4]. During fasting or illness, when glucose stores are depleted, the lack of alternative energy sources leads to symptoms like hypoglycemia, vomiting, dehydration, lethargy, poor feeding, developmental delays in infants, seizures, and other neurological issues [5,6,7]. Persistent and intractable metabolic acidosis has also been observed [8,9]. Several cases have even resulted in early death (before two years of age) [9,10,11]; however, other cases have remained asymptomatic [10,12]. HMGCS2D has an estimated incidence of <1/1,000,000 [13].

The diagnosis of HMGCS2D primarily relies on an HMG-CoA synthase enzyme assay; however, distinguishing between mitochondrial HMG-CoA synthase and cytosolic HMG-CoA synthase in liver homogenates is challenging, limiting the use of this assay [13]. Additionally, other biochemical indicators such as hypoketosis, elevated free fatty acids, normal acylcarnitines, ketonuria, and dicarboxyluria are not specific to HMGCS2D alone [7]. Recently, biomarkers like 4-hydrox-6-methyl-2-pyrone have been identified for HMGCS2D [14], but this is not part of routine laboratory testing. These factors highlight the diagnostic challenges of HMGCS2D, especially in asymptomatic patients during non-stress conditions due to its clinical overlap with other metabolic disorders and the absence of reliable biochemical markers [7,15]. As a result, genetic testing has become the definitive diagnostic tool for HMG-CoA synthase deficiency, utilizing methods such as Sanger sequencing, targeted next-generation sequencing, and exome sequencing [11,13,15].

The HMG-CoA enzyme is encoded by the *HMGCS2* gene (OMIM #600234), located on chromosome 1p12, consisting of 10 exons with distinct domains and features [11]. Pathogenic variants in the *HMGCS2* gene lead to the partial or complete loss of the HMG-CoA enzyme function, resulting in HMGCS2D. Since the first reported case in 1997, more than 60 cases with around 40 different *HMGCS2* pathogenic variants have been documented [11]. The majority of pathogenic variants are found in exons 2 and 4 [11,14,16]. The c.1201G>T (p.E401*) variant is the most common mutation identified in Chinese patients [11], while the c.634G>A (p.G212R) variant is more prevalent among individuals of European descent [14,17,18]. A genotype–phenotype correlation has been observed in HMGCS2D, where patients with biallelic truncation mutations tend to present with more severe clinical symptoms and exhibit a higher mortality rate [11].

In this study, we describe the clinical, biochemical, and molecular characteristics, along with the management strategies, of 19 cases from 14 unrelated families diagnosed with HMGCS2D.

## 2. Results

### 2.1. Clinical and Biochemical Characteristics

Nineteen cases of HMGCS2D from 14 unrelated families were diagnosed between October 2018 and October 2024 at the Center of Endocrinology, Metabolism, Genetic/Genomics, and Molecular Therapy, Vietnam National Children’s Hospital. Five families had siblings affected by HMGCS2D: P1 and P2, P3 and P4, P10 and P11, P13 and P14, and P16 and P17. Among these cases, 16 were symptomatic, while 3 (P4, P11, and P17) were asymptomatic, as shown in Table 1. The age of onset for the first acute episode ranged from 10 days to 28 months. Triggers for the initial crisis in the sixteen symptomatic cases included poor feeding (15/16, 93.8%), vomiting (9/16, 56.3%), diarrhea (4/16, 25.0%), and fever (3/16, 18.8%). The primary clinical features during the first episode were lethargy or coma (13/16, 81.3%), rapid breathing (11/16, 68.8%), hepatomegaly (9/16, 56.3%), shock (6/16, 37.5%), and seizures (3/16, 18.8%).

Hypoglycemia was observed in nine cases, with a median blood glucose level of 2.2 mmol/L (ranging from 0.1 to 2.48 mmol/L) (Table 2). Notably, patient P10 exhibited the highest level of hyperglycemia, which required insulin infusion. Metabolic acidosis was present in 12 of the 16 symptomatic cases, with a median pH of 7.18 (Table 2). Four cases—P8, P13, P14, and P16—did not exhibit metabolic acidosis. Elevated plasma ammonia levels were detected in five cases (25%), specifically in P6, P7, P10, P16, and P18. Severe hyperammonemia was recorded in P7, with a plasma ammonium concentration of 983 µmol/L. All 16 symptomatic cases had elevated plasma transaminase levels. Plasma creatine kinase was assessed in seven cases, and elevated levels (ranging from 297 to 3267 UI/L) were found in four patients: P3, P8, P14, and P16. Additionally, seven cases showed low free carnitine (plasma C0) levels, while only one case exhibited a high plasma C2 level. Dicarboxylic aciduria was identified in two cases, and ketonuria was detected in five cases.

**Figure 1:**
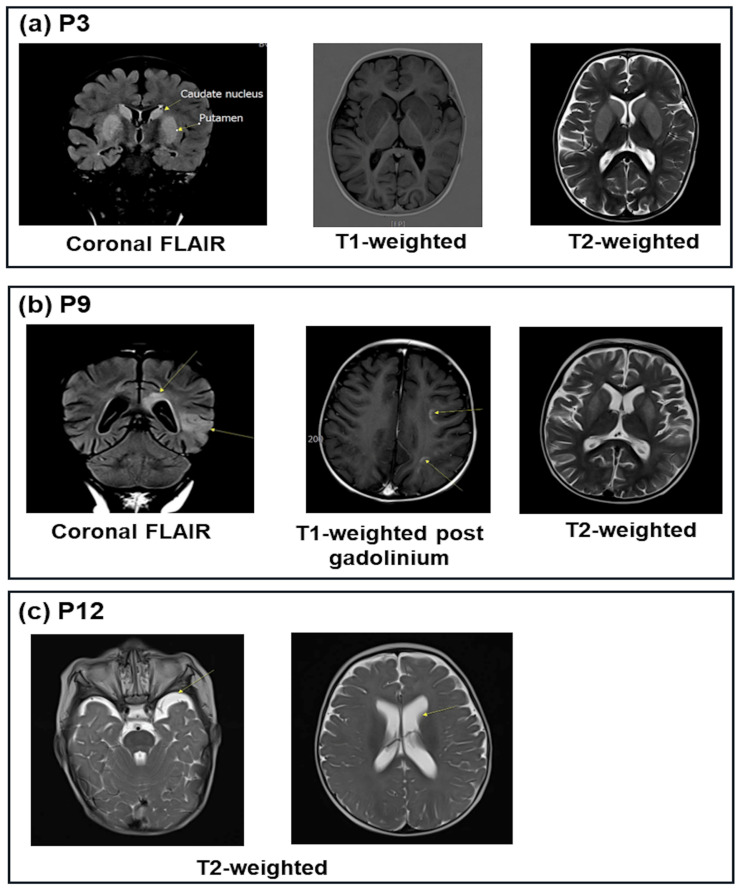
Magnetic resonance imaging (MRI) of three Vietnamese cases with mitochondrial 3-hydroxy-3-methylglutaryl-CoA synthase deficiency (HMGCS2D). (**a**) P3: coronal FLAIR, axial T1-weighted, and axial T2-weighted images showed symmetric hyperintensities involving both the head of caudate nuclei and putamina. (**b**) P9: coronal FLAIR image showed bilateral hyperintensities of periventricular deep white matter and left parietal cortex; axial T1-weighted post-gadolinium image showed enhancement of the subcortical area in the left frontal and parietal lobe; axial T2-weighted image showed bilateral symmetric hyperintensities involving putamen. (**c**) P12: axial T2-weighted MR image showing bilateral ventricular dilatation and the widening of temporal subarachnoid space predominant on the left side.

Brain magnetic resonance imaging (MRI) was performed in 11 cases with patients who presented with a coma during the acute crisis, and 3 of these cases exhibited brain abnormalities (Figure 1). Case P3 presented with symmetric hyperintensities in both the head of the caudate nuclei and the putamina (Figure 1a). Case P9 showed bilateral hyperintensities in the periventricular deep white matter and the left parietal cortex, along with enhancement in the subcortical regions of the left frontal and parietal lobes (Figure 1b). Case P12 demonstrated bilateral ventricular dilation and the widening of the temporal subarachnoid space, predominantly on the left side (Figure 1c).

### 2.2. Molecular Analyses

**Figure 2:**
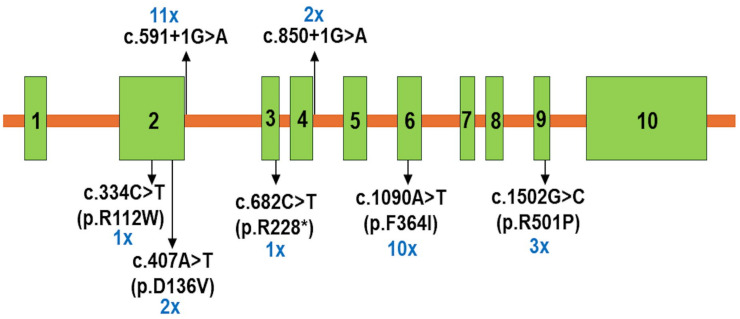
Scheme of the distribution of *HMGCS2* variants in 19 Vietnamese patients with mitochondrial HMG-CoA synthase deficiency.

**Figure 3:**
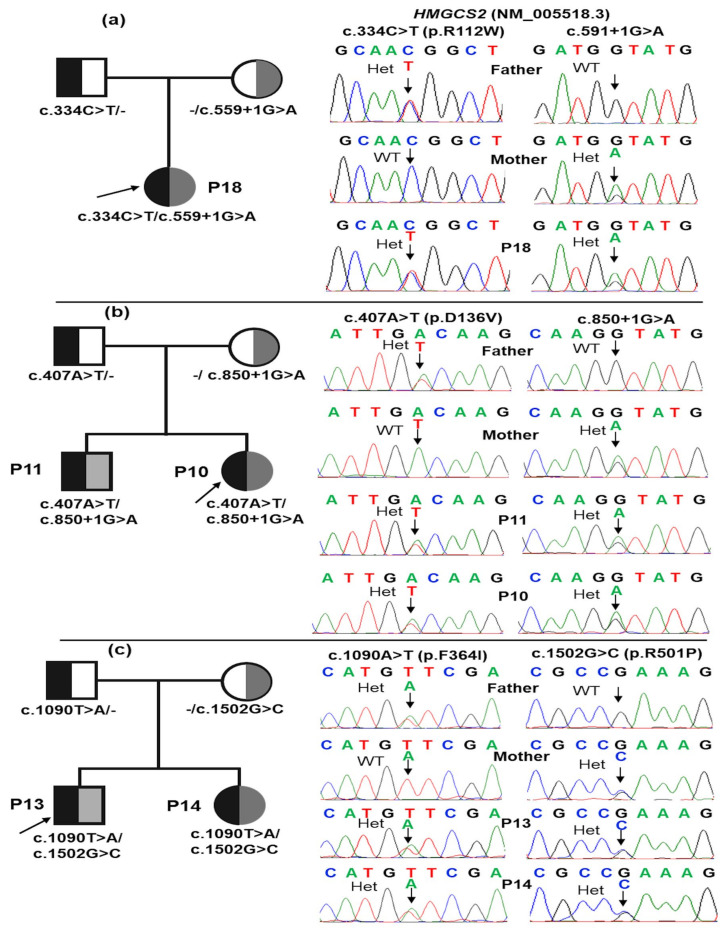
Pedigree and Sanger sequencing chromatograms of *HMGCS2* variants in the three families of the study. Rectangles and circles represent males and females; “-/” represents the normal allele; arrows indicate the probands; partially filled symbols indicate carrier parents; the filled symbol represents affected individuals; Het, heterozygous; WT, wild type. Patient P18 inherited c.334C>T (p.R112W) from her father and c.591+1G>A from her mother (**a**). Patients P10 and P11 inherited c.407A>T (p.D136V) from their father and c.850+1G>A from their mother (**b**). Patients P13 and P14 inherited c.1090T>A (p.F364I) from their father and c.1502G>C (p.R501P) from their mother (**c**).

A total of seven deleterious *HMGCS2* gene variants were identified in 19 patients with HMGCS2D, consisting of one nonsense variant, two splicing variants, and four missense variants (Table 3 and Figure 2). Homozygous variants were found in seven patients: P1, P2, P5, P7, P9, P16, and P17 (Table 3). The remaining 12 patients carried compound heterogeneous variants (Table 3). Among these, a novel variant, c.407A>T (p.D136V), has not been reported in any database. Five other variants—c.334C>T (p.R112W), c.559+1G>A, c.682C>T (p.R228*), c.1090T>A (p.F364I), and c.1502G>C (p.R501P)—have been classified as likely pathogenic or pathogenic in the ClinVar database. In contrast, the c.407A>T (p.D136V) and c.850+1G>A variants were not listed in ClinVar. According to the ACMG classification, c.407A>T (p.D136V) was categorized as a likely pathogenic variant, while c.850+1G>A was identified as a pathogenic variant (Table 3). Two common variants, c.559+1G>A and c.1090T>A (p.F364I), were detected in 11 out of 19 cases (57.9%) and 10 out of 19 cases (55.5%), respectively (Table 3 and Figure 2). Sanger sequencing in three families confirmed that patients P18, P10, P11, P13, and P14 inherited the mutant alleles from their parents (Figure 3).

### 2.3. Outcomes

Management of the first episode for the 16 symptomatic cases involved various interventions (Table 4). Mechanical ventilation was required in 11 cases for two to six days; acid-base correction was administered in 13 cases for 3 to 72 h; and glucose infusion was provided in 15 cases for one to seven days. Continuous veno-venous hemofiltration (CVVH) was performed in seven cases for 19 to 82 h. Antibiotic therapy was given to 14 cases for 2 to 28 days, and L-carnitine supplementation was administered in 13 cases. Additionally, patient P10 experienced hyperglycemia that required insulin infusion (0.05 UI/kg/h) to maintain normal blood glucose levels for four hours. Patient P7, who developed severe hyperammonemia (983 µmol/L), was treated with sodium benzoate supplementation. All patients recovered from the first episode, with the normalization of metabolic acidosis, transaminase, and CK levels. Only patient P3 experienced neurological sequelae, while the remaining patients showed normal mental and motor development. After discharge, 15 patients continued L-carnitine supplementation, and 2 of them also received arginine therapy.

It took between 10 days and 16 months to achieve an accurate diagnosis through molecular analysis (Table 5). A total of 14 patients were initially diagnosed with different inborn errors of metabolism as they presented with unknown severe metabolic acidosis/hypoglycemia/elevated transaminase and abnormal acylcarnitine/organic acid profiles. For example, patient 19’s organic acid profile revealed abnormalities of glutaric aciduria type 2. During the waiting period, seven patients experienced a total of eleven recurrent acute episodes between their first crisis and the confirmed diagnosis. Following genetic confirmation, all 19 patients were advised to avoid prolonged fasting. Sixteen symptomatic patients received management with L-carnitine and maltodextrin. Post-diagnosis care for all patients involved fasting avoidance, supplementation with L-carnitine and maltodextrin, glucose infusions, and carbohydrate-rich fluids during illnesses, effectively preventing further acute episodes. Only two recurrent acute attacks occurred in two patients after receiving accurate diagnosis and appropriate management. Currently, all 19 patients are alive, with ages ranging from 5 months to 14 years. All patients have normal physical development, and 18 out of 19 patients now show normal psychomotor development without any complications.

## 3. Discussion

Diagnosing HMGCS2D based on biochemical profiles presents important challenges. In our department, both the HMG-CoA synthase enzyme assay and specific urinary biochemical markers, such as 4-hydroxy-6-methyl-2-pyrone (4-HMP), are unavailable. Wu et al. suggested that a high C2 to C0 ratio, combined with urinary dicarboxylic acids during episodes of acute hypoglycemia and metabolic acidosis, could serve as an additional biochemical marker for HMGCS2D [11]. However, none of our patients fully met these criteria. For example, patient P16 exhibited a high C2/C0 ratio (7.5), urinary dicarboxylic acids, and hypoglycemia but did not present metabolic acidosis. Patient P19 had a high C2/C0 ratio (5.9), urinary dicarboxylic acids, and metabolic acidosis but maintained normal glucose levels. Notably, patient P13 only showed elevated transaminase levels. This variability in clinical and biochemical presentations during the first acute episode made it extremely difficult to promptly and accurately diagnose HMGCS2D in our patients. Initially, the 14 probands were misdiagnosed with various conditions, including organic acidurias, fatty acid oxidation disorders, and glycogen storage diseases. Our findings emphasize the essential role of genetic testing in accurately diagnosing HMGCS2D [7,10,11,12,19]. All patients in this study were confirmed to have biallelic mutations in the *HMGCS2* gene. Additionally, five siblings were diagnosed with HMGCS2D through family genetic screening. Among these five, three asymptomatic patients (P4, P11, and P17) displayed no clinical symptoms and maintained normal blood test results up to their current ages of 5 to 14 years. In contrast, their younger siblings experienced typical clinical manifestations between 5 and 14 months of age. Previous studies have reported only three asymptomatic cases [10,12]. The identification of our three asymptomatic patients further expands the database of asymptomatic individuals with HMGCS2D.

The median age of the first acute episodes in our study was seven months (ranging from 10 days to 28 months), which is slightly younger than the median age of nine months reported in other studies [11]. Notably, one patient (P7) experienced an acute episode at just 10 days old despite having no prior digestive issues or infections. However, the patient’s mother had a history of polyhydramnios and gestational diabetes starting at 24 weeks of gestation, which required insulin treatment, and the infant’s birth weight was 3900 g. These factors—gestational diabetes, polyhydramnios, and high birth weight—may have contributed to neonatal hypoglycemia, potentially triggering the acute episode. Additionally, the initial acute episodes in our patients often followed digestive symptoms such as poor feeding (15 out of 16 cases), vomiting (9 out of 16), and diarrhea (four out of 16), which is consistent with findings from other studies [10,11]. The onset of HMGCS2D typically occurs in infancy, often after digestive disturbances that impair nutrient absorption and increase catabolic activity, leading to enhanced gluconeogenesis and reliance on ketone bodies for energy. The clinical symptoms observed in the 16 symptomatic patients during acute episodes included poor appetite, vomiting, diarrhea, fever, rapid breathing, lethargy or coma, convulsions, and shock. These symptoms closely resemble those of sepsis or acute brain syndrome, making diagnosis particularly challenging [20,21].

Hypoglycemia is considered one of the diagnostic indicators for HMGCS2D; however, it is not a defining feature during metabolic crises, as supported by previous studies [17,22] and confirmed by our study findings. In our study, 4 out of 16 symptomatic patients maintained normal glucose levels during episodes of metabolic decompensation. Additionally, patient P10 presented with considerable hyperglycemia (24.5 mmol/L) accompanied by metabolic acidosis, ketonuria, and elevated transaminase levels on the first day of the acute episode, closely resembling diabetic ketoacidosis. Similar instances of hyperglycemia in HMGCS2D have been reported in a patient from Thailand [16], one from Turkey [10], and one from the United States [19]. The etiology of this dysglycemia in HMGCS2D remains unclear.

Additionally, brain damage detected through MRI during acute episodes was observed in 3 out of 11 examined patients. The brain lesions were located in the bilateral putamen and caudate nuclei, as well as in the white matter of the cerebral cortex, along with dilated lateral ventricles. These findings are consistent with brain abnormalities reported in patients with HMGCS2D in previous studies [7,11,17].

In this study, the variants c.559+1G>A and c.1090T>A (p.F364I) were identified as the two most common mutations in Vietnamese patients with HMGCS2D, each occurring in more than 50% of cases. The c.559+1G>A variant affects the 5′ splice site of intron 2 in the *HMGCS2* gene and is predicted to disrupt the donor splice site, supported by a high Δ score of 0.97 in SpliceAI. This disruption likely interferes with the normal splicing process of the *HMGCS2* gene. This variant has been reported in the population database dbSNP155 (rs587603096) and has been submitted as a pathogenic variant by one laboratory to ClinVar (ID: 859738). Similar splice site disruptions have been observed in other individuals with HMGCS2D [11,12]. The second most common variant, c.1090T>A, results in the substitution of phenylalanine with isoleucine at position 364 (p.F364I) of the HMGCS2 protein. Phenylalanine contains an aromatic side chain, whereas isoleucine has a branched hydrocarbon side chain. This amino acid change may alter the protein’s side chain structure, potentially affecting the conformation and function of the HMGCS2 protein. Additionally, the c.1502G>C (p.R501P) variant was found in three patients and has been previously reported to be a common pathogenic variant in Thai patients with HMGCS2D [16]. Arginine 501 (R501) has been predicted to be a critical amino acid for the proper function of the HMGCS2 protein, and the R501P substitution leads to the loss of a salt bridge between arginine 501 and aspartic acid 101 [23]. This structural disruption considerably impacts the protein’s stability and function. Furthermore, functional assays of the R501P mutation demonstrated a complete loss of HMGCS2 enzymatic activity [23]. The fourth identified variant, c.334C>T (p.R112W), was previously reported in a patient of Romanian origin [15]. Although the mutated p.R112W protein showed moderate expression levels (22.4%) compared to the wild-type protein, enzyme activity assays revealed a complete absence of enzymatic function in the mutant protein [15]. The fifth variant, a nonsense mutation c.682C>T (p.R228*), has not been previously reported in the medical literature in individuals with HMGCS2-related disorders. However, this variant has been submitted as a pathogenic variant by one laboratory to ClinVar (ID: 2080601). The c.682C>T mutation introduces a premature termination codon at residue 228 (p.Arg228*) of the HMGCS2 protein, resulting in partial deletion or the absence of the HMGCS2 protein product.

**Figure 4:**
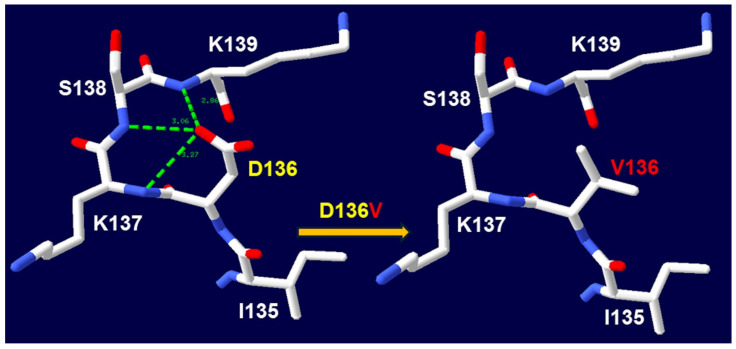
The D136V change in the three-dimensional structure of human mitochondrial 3-hydroxy-3-methylglutaryl-coenzyme a synthase 2 (PDB ID: 2WYA). The wildtype is D136 and the mutant is V136. The D136V change causes H-bond losses between D136 and K137, S138, and K139.

Our study, to the best of our knowledge, is the first to report the association of the c.407A>T (p.D136V) and c.850+1G>A variants with HMGCS2D. The sixth variant, c.407A>T, results in the substitution of aspartic acid at position 136 with valine. This D136V mutation disrupts three hydrogen bonds between D136 and the neighboring amino acids K137, S138, and K139 (Figure 4), leading to an unstable HMGCS2 protein structure. The seventh variant, c.850+1G>A, is located at the 5′ splice site of intron 5 in the *HMGCS2* gene. This mutation is predicted to disrupt the canonical donor splice site, supported by a high Δ score of 0.99, indicating its significant impact on proper splicing. Although this variant is listed in the dbSNP database (rs112412189), it has not previously been reported in individuals affected by HMGCS2-related disorders.

According to a previous review publication [11], 6 out of 49 patients with HMGCS2D died due to hypoglycemic crises and severe metabolic acidosis during acute episodes. HMGCS2D impairs the body’s ability to produce ketone bodies and regulate energy metabolism, particularly during periods of fasting or illness. Therefore, high glucose infusion and proactive management—such as preventing prolonged fasting and providing enteral carbohydrates or glucose infusion during illness—are essential for effective treatment. This management approach considerably reduced the acute relapse rate in our patients, decreasing from 11 to 2 recurrent crises after an accurate diagnosis. Symptomatic patients were treated with L-carnitine supplementation, frequent meals, and maltodextrin. To date, none of the patients in our study have died, and only one patient has experienced neurological sequelae.

The limitations of our study included a small sample size and a lack of functional evidence. The effect of the variants on the protein function needs to be investigated by further experimental work, especially in symptomatic patients and asymptomatic siblings, to explore the mechanism of this situation.

## 4. Materials and Methods

### 4.1. Individuals

This study included both retrospective and prospective analyses of 19 Vietnamese children from 14 unrelated families diagnosed with HMGCS2D at the Center of Endocrinology, Metabolism, Genetic/Genomics, and Molecular Therapy, Vietnam National Children’s Hospital.

### 4.2. Clinical Characteristics

Clinical symptoms such as fever, vomiting, diarrhea, poor feeding, breathing difficulty, seizures, hepatomegaly, and lethargy were closely monitored and documented. Urinary organic acid analysis was conducted using Gas Chromatography–Mass Spectrometry (GC/MS; Shimadzu model QP 5000, Shimadzu, Kyoto, Japan) following the method described in a previous study [24]. Acylcarnitine analysis was performed using Guthrie blotting paper [25,26]. Dried blood samples were automatically punched into 3.2 mm diameter circles with an automatic puncher (WallacAutoPuncher™; PerkinElmer, Waltham, MA, USA). Three 3.2 mm diameter circles were placed in an Amicon Ultra 0.5 (10K) tube containing 100 µL of distilled water and incubated at 37 °C for 2 h with occasional shaking. The mixture was then centrifuged at 12,500 rpm for 15 min. The resulting extracted solution was transferred to a new tube, and 110 μL of stable-isotope internal standards in methanol was added for further analysis. The isotope standards used in the analysis included 2 nmol of glycine-^2^H_2_; 1.5 nmol each of alanine-^2^H_3_, valine, and leucine-^2^H_10_; 0.5 nmol each of methionine-^2^H_3_, phenylalanine-^2^H_5_, and arginine-13C6; 0.8 nmol of tyrosine-^2^H_2_; 0.2 nmol of citrulline-^2^H_3_; 3 nmol of glutamine-^2^H_5_; 100 pmol of carnitine-^2^H_3_; 100/3 pmol of acetyl-carnitine-^2^H_3_; 50/3 pmol each of propionyL-carnitine-^2^H_3_ and glutaryL-carnitine-^2^H_9_; 10 pmol of butyryl-carnitine-^2^H_3_; and 20/3 pmol each of octanoyL-carnitine-^2^H_3_ and palmitoyl-carnitine-^2^H_9_. The mass spectrometry system used was a triple-stage mass spectrometer, Model TSQ7000 (Thermo-Quest, Tokyo, Japan), equipped with a Model LC10 HPLC system and a Model SIL-10ADVP autoinjector (Shimadzu, Kyoto, Japan). The autoinjector introduced the derivatized sample (13 μL) at a flow rate of 20 μL/s using 50% acetonitrile in water at 1.9 min intervals. The resolution of the mass spectra was automatically adjusted, and data were collected in the receiving channels for 1.2 min per sample. Images for amino acid and acylcarnitine analysis were captured, and peak data were automatically processed using Analyst 1.5.1 software (AB SCIEX, Foster City, CA, USA).

### 4.3. Genetic Analysis

Genomic DNA was extracted from whole-blood samples using the QIAamp DNA Blood Kit (Qiagen, Hilden, Germany). Genetic testing was conducted at Invitae (Invitae Corporation, San Francisco, CA, USA) and the Center for Gene and Protein Research, Hanoi Medical University using a comprehensive glycogen storage disease and fatty acid oxidation defects panel that included 46 genes comprising the following: *ACADM* (NM_000016.5), *ACADS* (NM_000017.3), *ACADSB* (NM_001609.3), *ACADVL* (NM_000018.3), *AGL* (NM_000642.2), *ALDOA* (NM_000034.3), *CPT1A* (NM_001876.3), *CPT2* (NM_000098.2), *ENO3* (NM_053013.3), *ETFA* (NM_000126.3), *ETFB* (NM_001985.2), *ETFDH* (NM_004453.3), *FBP1* (NM_000507.3), *G6PC* (NM_000151.3), *GAA* (NM_000152.3), *GBE1* (NM_000158.3), *GYG1* (NM_004130.3), *GYS1* (NM_002103.4), *GYS2* (NM_021957.3), *HADH* (NM_005327.4), *HADHA* (NM_000182.4), *HADHB* (NM_000183.2), *HMGCL* (NM_000191.2), *HMGCS2* (NM_005518.3), *LAMP2* (NM_002294.2), *LDHA* (NM_005566.3), *MLYCD* (NM_012213.2), *NADK2* (NM_001085411.2), *PFKM* (NM_000289.5), *PGAM2* (NM_000290.3), *PGM1* (NM_002633.2), *PHKA1* (NM_002637.3), *PHKA2* (NM_000292.2), *PHKB* (NM_000293.2)*, PHKG2* (NM_000294.2)*, POLG* (NM_002693.2)*, PYGL* (NM_002863.4), *PYGM* (NM_005609.3), *RBCK1* (NM_031229.3), *SLC22A5* (NM_003060.3), *SLC25A20* (NM_000387.5), *SLC2A2* (NM_000340.1), *SLC37A4* (NM_001164277.1), *SLC52A1* (NM_017986.3), *SLC52A2* (NM_024531.4), and *SLC52A3* (NM_033409.3).

The pathogenicity of the identified variants was predicted using Mutation Taster [27]. The American College of Medical Genetics and Genomics (ACMG) and the Association for Molecular Pathology (AMP) guidelines were used for variant classification [28]. The splicing variants were further analyzed in silico using the SpliceAI tool (https://spliceailookup.broadinstitute.org, accessed on 16 September 2023) [29]. To confirm the identified variants, exons 2, 3, 4, 6, and 9 of the *HMGCS2* gene were amplified using specifically designed oligonucleotide primers (Table 6). The PCR products were sequenced using the 3500 Genetic Analyzer capillary electrophoresis system (Life Technologies, Foster City, CA, USA). The reference sequence used for *HMGCS2* was NM_005518.3. Additionally, the impact of the variants on the three-dimensional structure of the human mitochondrial 3-hydroxy-3-methylglutaryl-coenzyme A synthase 2 (HMGCS2, PDB ID: 2WYA) was modeled using the Swiss-PdbViewer version 4.1.0 [30].

### 4.4. Management

The patients were initially admitted to the hospital with the misdiagnoses of other inborn errors of metabolism or unknown elevated transaminase levels. Acute management included the administration of a 10% dextrose infusion with a glucose delivery rate of 8–10 mg/kg/min, the prompt initiation of oral feeding, when possible, the correction of acidosis using sodium bicarbonate or continuous veno-venous hemofiltration (CVVH), supplementation with L-carnitine, and the treatment of triggering factors, such as antibiotics for infections and antipyretic drugs for fever. Proactive management strategies focused on preventing fasting. For infants aged 0–4 months, feeding was scheduled every three hours, and for infants older than 4 months and up to 12 months, fasting was limited to no more than eight hours. Beyond infancy, regular feeding schedules were maintained. During illness, patients were aggressively managed with oral or enteral carbohydrate-rich fluids every 3–4 h, along with 10% dextrose infusion at 1.5 times the maintenance rate during periods of illness, poor oral intake, or preoperative fasting. L-carnitine supplementation was also continued to support energy metabolism.

## 5. Conclusions

We have described 16 symptomatic and 3 asymptomatic Vietnamese patients diagnosed with HMG-CoA synthase deficiency. Seven pathogenic or likely pathogenic *HMGCS2* variants were identified, including two common variants, c.559+1G>A and c.1090T>A, as well as a novel variant, c.407A>T. Following an accurate diagnosis, proactive management was implemented, resulting in a reduction in the acute relapse rate. All patients are currently alive and exhibit normal physical development.

## Figures and Tables

**Table 1:** Clinical symptoms of 19 patients with mitochondrial 3-hydroxy-3-methylglutaryl-CoA synthase deficiency.

Pt	Age of Onset	Gender	Family History	Days of Onset	Fever	Vomiting	Diarrhea	Poor Feeding	Rapid Breathing	Seizure	Hepatomegaly	Lethargy/ Coma	Others
P1	5 m	M	+	3.0	-	-	-	+	+	-	+	+	Shock
P2	7 m	F	+	1.0	+	+	-	+	+	-	-	+	Shock
P3	14 m	M	+	1.0	-	+	+	+	+	-	+	+	Shock
P4	-	M	+	-	-	-	-	-	-	-	-	-	-
P5	18 m	M	-	2.0	+	-	-	+	+	-	+	+	Shock
P6	8 m	F	-	1.0	-	+	-	+	+	-	+	+	Shock
P7	10 d	F	-	1.0	-	-	-	+	+	-	NA	+	Jaundice
P8	28 m	M	-	5.0	-	+	+	+	+	+	+	+	Pallor
P9	13 m	M	-	4.0	-	+	-	+	+	+	+	+	-
P10	8 m	F	+	1.0	-	-	-	+	-	-	+	+	Shock
P11	-	M	+	-	-	-	-	-	-	-	-	-	-
P12	7 m	F	-	3.0	-	+	+	+	+	-	+	+	-
P13	18 m	M	+	1.0	-	-	+	+	-	-	+	-	-
P14	5 m	F	+	0.5	-	+	-	+	-	-	-	-	-
P15	6 m	F	-	2.0	NA	NA	NA	NA	NA	NA	NA	NA	NA
P16	5 m	F	+	2.0	-	-	-	+	-	+	NA	+	-
P17	-	M	+	-	-	-	-	-	-	-	-	-	-
P18	10 m	F	-	2.0	-	+	-	+	+	-	NA	+	-
P19	6 m	F	-	2.0	+	+	-	+	+	-	-	+	Loss weight

M, male; F: female; NA, not analyzed; +, present; -, not present; m, month; d, day. P1 and P2 are siblings; P3 and P4 are siblings; P10 and P11 are siblings; P13 and P14 are siblings; P16 and P17 are siblings. Three cases, P4, P11, and P17, were asymptomatic and diagnosed through family screening.

**Table 2:** Biochemical features of 16 symptomatic cases in the first episode.

Pt	Blood Glucose mmol/L	Metabolic Acidosis			Plasma Ammoniac μmol/L	ALT UI/L	AST UI/L	CK IU/L	Plasma C0 µmol/L	Plasma C2 µmol/L	Urinary Dicarboxylic Acid	Ketonuria	Brain MRI
		pH	HCO3 mmol/L	BE									
P1	↓2.0	**6.96**	**4.2**	**−27.0**	98.4	↑88	↑138	NA	13.2	8.5	-	1+	Normal
P2	↓1.1	**7.25**	**5.3**	**−22.0**	64.2	↑62	↑112	126.8	14.9	9.5	-	Neg	Normal
P3	↓0.8	**7.12**	**9.6**	**−21.0**	51.6	↑108	↑209	↑2287	↓4.4	27.0	-	1+	Lesions of the bilateral putamen caudate
P5	4.8	**7.30**	**5.9**	**−20.0**	38.4	↑212	↑159	NA	9.1	9.5	-	3+	Normal
P6	↓1.8	**6.99**	**4.6**	**−27.0**	↑109.2	↑83	↑290	NA	↓5.0	4.6	-	Trace	NA
P7	3.9	**6.86**	**2.7**	**−29.0**	↑983.0	↑56	↑139	NA	↓5.0	4.7	-	Neg	NA
P8	↓0.1	7.47	25.2	0.5	37.2	↑133	↑148	↑297	↓6.0	20.4	-	Neg	Normal
P9	3.82	**7.00**	**8.6**	**−20.0**	49.2	↑126	↑143	NA	16.7	8.8	-	Neg	Bilateral cerebrum lesions
P10	↑25.45	**6.89**	**1.5**	**−31.0**	↑156.6	↑57	↑222	NA	6.9	27.3	-	Trace	Normal
P12	↓2.48	**7.37**	**7.5**	**−15.6**	36.4	↑132	↑270	NA	↓3.8	19.7	-	1+	Slight ventilation dilation
P13	4.5	7.40	21.0	−4.0	31.8	↑465	↑691	↑1198	10.1	22.5	-	Neg	NA
P14	4.46	7.39	21.5	−3.0	52.4	↑56	↑97	67	26.2	11.1	-	Neg	NA
P15	↓2.4	**7.05**	**3.2**	**−24.0**	71.0	↑105	↑280	NA	↓3.3	18.5	-	Neg	NA
P16	↓0.9	7.41	23.0	NA	↑244.0	↑56	↑90	↑3267	8.4	↑62.8	+	Neg	Normal
P18	↓2.08	**7.10**	**3.0**	**−21.0**	↑226.0	32	↑84	NA	12.2	14.7	-	Neg	Normal
P19	3.65	**7.27**	**4.5**	**−21.0**	43.8	↑131	↑121	59	↓3.8	22.3	+	Neg	Normal

BE, base excess; AST, aspartate aminotransferase; ALT, alanine aminotransferase; CK, creatine kinase; ↓, low level; ↑, elevated level, -, none; +, yes; Neg, negative; 1+, positive with ketone level < 1.5 mmol/L; 3+, positive with 3.9 mmol/L ≤ ketone level < 7.8 mmol/L; NA, not analyzed; MRI, magnetic resonance imaging. In the metabolic acidosis columns, the bold font indicates metabolic acidosis.

**Table 3:** Molecular findings in 19 Vietnamese patients with HMGCS2D.

Patients	Genotype		AA Change	Effect	Mutation Taster	dbSNP	ClinVar	ACMG Classification
06 (P1, P2, P7, P9, P16, P17)	Hom	c.559+1G>A		Splicing	Deleterious	rs587603096	Pathogenic 859738	Pathogenic (PVS1, PM1, PM2, PM3, PP1, PP3, PP4, and PP5)
05 (P3, P4, P6, P8, P12)	CH	c.559+1G>A		Splicing	Deleterious	rs587603096	Pathogenic 859738	Pathogenic (PVS1, PM1, PM2, PM3, PP1, PP3, PP4, and PP5)
		c.1090T>A	F364I	Missense	Deleterious	rs1652807016	Pathogenic 859739	Likely pathogenic (PM1, PM2, PM3, PP1, PP3, PP4, and PP5)
01 (P5)	Hom	c.1090T>A	F364I	Missense	Deleterious	rs1652807016	Pathogenic 859739	Likely pathogenic (PM1, PM2, PM3, PP1, PP3, PP4, and PP5)
02 (P10, P11)	CH	c.407A>T	D136V	Missense	Deleterious			Likely pathogenic (PM1, PM2, PM5, PP1, PP3, and PP4)
		c.850+1G>A		Splicing	Deleterious	rs112412189		Pathogenic (PVS1, PM2, PM3, PP1, PP3, and PP4)
03 (P13, P14, P15)	CH	c.1090T>A	F364I	Missense	Deleterious	rs1652807016	Pathogenic 859739	Likely pathogenic (PM1, PM2, PM3, PP1, PP3, PP4, and PP5)
		c.1502G>C	R501P	Missense	Deleterious	rs372079931	Pathogenic 452101	Pathogenic (PS3, PM1, PM2, PM3, PP1, PP3, PP4, and PP5)
01 (P18)	CH	c.682C>T	R228*	Nonsense	Deleterious	rs763531478	Pathogenic 2080601	Pathogenic (PVS1, PM2, PM3, PP3, and PP4)
		c.1090T>A	F364I	Missense	Deleterious	rs1652807016	Pathogenic 859739	Likely pathogenic (PM1, PM2, PM3, PP1, PP3, PP4, and PP5)
01 (P19)	CH	c.334C>T	R112W	Missense	Deleterious	rs768707273	Likely pathogenic SCV003828997.2	Likely pathogenic (PM1, PM2, PM3, PP3, and PP4)
		c.559+1G>A		Splicing	Deleterious	rs587603096	Pathogenic 859738	Pathogenic (PVS1, PM1, PM2, PM3, PP1, PP3, PP4, and PP5)

AA: amino acid; Hom, homozygous; CH, compound heterozygous; ACMG, American College of Medical Genetics and Genomics; PVS, pathogenic very strong; PM, pathogenic moderate; PP, pathogenic support.

**Table 4:** Management and outcome at the first episode.

Pt	Initial Diagnosis	Management in First Crisis						Outcome of First Crisis		
		Ventilation (Days)	Acidotic Correction (h)	Glucose Infusion (Days)	CVVH (h)	Antibiotics (Days)	Others	Recovered MA (h)	Alive	Neurological
P1	Organic aciduria	3	34	4	34	13	L-carnitine, biotin, B12	13	Yes	No
P2	HMGCS2D	2	48	2	19	10	L-carnitine	48	Yes	No
P3	Glutaric acidemia II	5	26	3	20	20	L-carnitine, B2, coenzyme Q10	11	Yes	Yes
P5	MMA/GA II	3	33	6	0	16	L-carnitine, B2, B12, biotin	33	Yes	No
P6	Myocarditis/IEM	4	72	6	82	28	L-carnitine	72	Yes	No
P7	Glutaric acidemia II	2	48	7	0	19	L-carnitine	48	Yes	No
P8	Hypoglycemia/IEMs	0	24	4	0	0	L-carnitine	24	Yes	No
P9	IEMs	6	48	4	0	21	0	48	Yes	No
P10	Glycogen storage	2	25	2	32	15	L-carnitine, biotin, B12	14	Yes	No
P12	CUD	0	18	2	0	12	L-carnitine	18	Yes	No
P13	Elevated transaminase	0	0	0	0	7	0	0	Yes	No
P14	HMGCS2D	0	0	1	0	0	L-carnitine	0	Yes	No
P15	IEM	3	0	3	48	3	L-carnitine,	72	Yes	No
P16	FAOD	3	3	1	0	2	L-carnitine, biotin, B12	48	Yes	No
P18	IEM	3	3	3	72	7	L-carnitine, B12, arginine, biotin	72	Yes	No
P19	Glutaric aciduria II	0	10	5	0	9	L-carnitine, B2, coenzyme Q10	48	Yes	No
Sum		11/16	13/16	15/16	7/16	14/16	14/16			

CVVH, continuous veno-venous hemofiltration; HMGCS2D, mitochondrial HMG-CoA synthase deficiency; MMA/GA II, methylmalonic acidemia/glutaric acidemia II; IEM, inborn errors of metabolism; CUD, carnitine uptake defect; FAOD, fatty acid oxidation disorders.

**Table 5:** Diagnosis and follow–up.

Pt	Age of AD	Time to Achieve AD	Management Before AD	Crisis Numbers Before AD	Current Age	Crisis Numbers After AD	Height (SDS)	Weight (SDS)	DQ
P1	21 m	16 m	L-carnitine, biotin, B12	4 (2 times: CVVH)	7 y 2 m	1	−0.2	−0.4	Normal
P2	7 m	10 d	L-carnitine	1	1 y 11 m	0	−0.2	0.2	Normal
P3	17 m	3 m	L-carnitine	1	4 y	0	NA	−2.1	Neurological sequelae
P4	6 y			0	9 y 8 m	0	0.1	0.7	Normal
P5	26 m	8 m	L-carnitine	0	7 y 8 m	0	1.2	1.7	Normal
P6	9 m	1 m	L-carnitine	1	4 y 3 m	0	1.1	0.8	Normal
P7	1 m	1 m	L-carnitine	0	2 y 5 m	0	0.8	1.2	Normal
P8	3 y 3 m	11 m	L-carnitine	2	7 y 3 m	0	−0.8	−1.1	Normal
P9	17 m	4 m	L-carnitine	0	3 y	0	−0.5	−1.6	Normal
P10	10 m	2 m	L-carnitine	0	2 y 5 m	0	−1.0	−0.4	Normal
P11	12 y			0	14 y 1 m	0	0.2	−1.2	Normal
P12	8 m	1 m	L-carnitine	0	13 m	0	1.4	0.7	Normal
P13	19 m	1 m	Arginine, vitamin E, L-carnitine	0	3 y 4 m	0	1.7	0.7	Normal
P14	5 m			0	5 m	1	3.1	−0.9	Normal
P15	10 m	4 m	Arginine, L-carnitine	1 (CVVH)	18 m	0	−0.8	−0.8	Normal
P16	8 m	3 m	L-carnitine, biotin, vitamin B12	1	14 m	0	0	0	Normal
P17	4 y 6 m			0	5 y	0	0.3	−0.2	Normal
P18	12 m	2 m	L-carnitine	0	17 m	0	−0.9	−0.2	Normal
P19	9 m	3 m	L-carnitine, CoQ10, vitamin B2	0	19 m	0	0.1	−0.8	Normal

m, month; y, year; AD, accurate diagnosis; CVVH, continuous veno-venous hemofiltration diagnosis; DQ, development quotient.

**Table 6:** List of primers used in this study.

Gene	Exon	Sequence (5′–3′)
*HMGCS2*	2	F: GGTCTACTTCCCAGCCCAAT R: CACCTGGGGAACTGAAAAGC
*HMGCS2*	3	F: CCAGGACCTAGAATTGTGCC R: CCCTCTGCTCCATAGACCAG
*HMGCS2*	4	F: GCTCAAGGTAGGCTGCATTG R: GGCTGACATCCCTTGGTTTC
*HMGCS2*	6	F: CCCCAAAGTCCTCTCCAGAA R: CCAACTTTGTTGACCCTGCA
*HMGCS2*	9	F: TTACAGCCCAGCCAAGAGAG R: TTCTCCTGTCACCCCAATCC

F, forward; R, reverse.

## Data Availability

The raw data supporting the conclusions of this article will be made available by the authors on request.
